# Comparison of Polymerase Chain Reaction–Restriction Fragment Length Polymorphism, Immunohistochemistry, and DNA Sequencing for the Detection of *IDH1* Mutations in Gliomas

**DOI:** 10.31557/APJCP.2020.21.11.3229

**Published:** 2020-11

**Authors:** Rusdy Ghazali Malueka, Emilia Theresia, Fitria Fitria, Ibnu Widya Argo, Aditya Dwi Donurizki, Sabillal Shaleh, Meutia Rizki Innayah, Adiguno Suryo Wicaksono, Kusumo Dananjoyo, Ahmad Asmedi, Rachmat Andi Hartanto, Ery Kus Dwianingsih

**Affiliations:** 1 *Department of Neurology, Faculty of Medicine, Public Health, and Nursing, Universitas Gadjah Mada, Dr. Sardjito General Hospital, Yogyakarta, Indonesia. *; 2 *Department of Anatomical Pathology, Faculty of Medicine, Public Health, and Nursing, Universitas Gadjah Mada, Dr. Sardjito General Hospital, Yogyakarta, Indonesia. *; 3 *Division of Neurosurgery, Department of Surgery, Faculty of Medicine, Public Health, and Nursing, Universitas Gadjah Mada, Dr. Sardjito General Hospital, Yogyakarta, Indonesia. *

**Keywords:** Glioma, IDH1 gene, DNA sequencing, PCR, RFLP, immunuhistochemistry

## Abstract

**Background:**

*IDH1* mutation shows diagnostic, prognostic, and predictive value in gliomas. Direct Sanger sequencing is considered the gold standard to detect *IDH1* mutation. However, this technology is not available in most neuropathological centers in developing countries such as Indonesia. Immunohistochemistry (IHC) and polymerase chain reaction–restriction fragment length polymorphism (PCR–RFLP) have also been used to detect *IDH1* mutation. This study aimed to compare DNA sequencing, IHC, and PCR–RFLP in detecting *IDH1* mutations in gliomas.

**Methods:**

Research subjects were recruited from Dr. Sardjito Hospital. Genomic DNA was extracted from fresh or formalin-fixed paraffin-embedded samples of tumor tissue. DNA sequencing, PCR–RFLP and IHC were performed to detect *IDH1* mutation. Sensitivity, specificity, and accuracy of PCR–RFLP and IHC were calculated by comparing them to DNA sequencing as the gold standard.

**Results:**

Among 61 recruited patients, 13 (21.3%) of them carried a mutation in codon 132 of the *IDH1* gene, as shown by DNA sequencing. PCR–RFLP and DNA sequencing have a concordance value of 100%. Meanwhile, the concordance value between *IDH1* R132H IHC and DNA sequencing was 96.7%. The sensitivity, specificity, positive predictive values, negative predictive values, and accuracy for PCR–RFLP were all 100%. On the other hand, the sensitivity, specificity, and accuracy of IHC were 92.3%, 97.9%, and 96.7%, respectively.

**Conclusion:**

This study showed that both PCR–RFLP and IHC have high accuracy in detecting *IDH1* mutation. We recommend a combination of PCR–RFLP and IHC to detect *IDH1* mutation in resource-limited settings.

## Introduction

Glioma is one of the most common primary brain tumors. Molecular characteristics are now an important part of glioma diagnosis and therapy. In 2016, the World Health Organization (WHO) even integrated these molecular characteristics into glioma classifications. One of the most important biomarkers in gliomas is mutation in the isocitrate dehydrogenase (IDH) gene (Louis et al., 2016). The *IDH1* gene is located in the chromosome region 2q33. This gene encodes isocitrate dehydrogenases enzymes, which convert isocitrate to α-ketoglutarate (Bujko et al., 2010). Most mutations in the *IDH1* gene happen in codon 132 of the gene. The most frequent mutation is G to A missense mutation at position 395 of the *IDH1* transcript (c.395G>A). This results in substitution of the amino acid arginine with histidine (R132H) (Agarwal et al., 2013). Approximately 90% of patients with *IDH1*/2 mutations carry this mutation. Other types of mutations in codon 132 of *IDH1* genes are much smaller in number. These mutations are c.394C> T (p.R132C) by 4%, c.394C> A (p.R132S) or c.394C> G (p.R132G) by 1.5% each, and c.395G> T (p.R132L) or c.394C> G + c.395G> T (p.R132 V) of less than 1% (Mellai et al., 2011). 

*IDH1* mutation has been shown to have diagnostic, prognostic, and predictive value with regard to gliomas. As a diagnostic marker, this mutation can be identified in 55–80% of grade II and III oligodendrogliomas and astrocytomas. *IDH1* mutations are more frequently observed in secondary GBM (>80%) compared with primary GBM (<10%). Glioma patients with IDH mutations have been shown to have better prognosis compared to patients with wild-type IDH (Goh et al., 2019; Uno et al., 2011; Van den Bent et al., 2010). Median overall survival (OS) of GBM patients with this mutation is 31 months, much longer than the median OS of 15 months found in wild-type patients (Fu et al., 2010; Kloosterhof et al., 2011; Lee et al., 2007). IDH mutations also predict response to the alkylating agent temozolomide. GBM patients with IDH mutations show better response to temozolomide administration. From the above explanation, it is clear that identification of IDH mutations is very important in glioma patients. Hence, IDH mutation testing is currently recommended as part of the standard diagnosis of gliomas (Krell et al., 2013). 

IDH mutation assessment can be done with DNA-based tests or immunohistochemical tests to detect mutated proteins (Preusser et al., 2011). The most frequently performed tests to detect IDH mutations are immunohistochemistry (IHC) and Sanger sequencing (Zou et al., 2015). The algorithm currently recommended for IDH mutation detection is *IDH1* R132H mutation identification using IHC, followed by DNA-based examination (PCR and sequencing) if IHC shows negative results. This DNA-based examination is expected to detect other *IDH1* mutations that are less frequent (Catteau et al., 2014). IHC using specific antibodies for *IDH1* R132H mutation has been shown to have high sensitivity and specificity. Previous studies have also reported good concordance of immunostaining and DNA-sequencing (Catteau et al., 2014; Preusser et al., 2011). However, IHC examination is often subjective and sometimes has problems due to the presence of background staining or regional heterogeneity of the mutant protein expression (Catteau et al., 2014; Preusser et al., 2011). 

Regarding DNA-based analyses, direct Sanger sequencing is considered the gold standard for the detection of *IDH1* mutation. However, this technology is labor intensive, requiring sophisticated equipment and trained personnel, and not readily available in all neuropathological centers (Agarwal et al., 2013; Catteau et al., 2014). 

As explained earlier, the majority of *IDH1* mutations occur in codon number 132 from the *IDH1* transcript. This opens up opportunities for simple test applications based on restriction digestion (Bujko et al., 2010; Elsayed et al., 2014). Polymerase chain reaction–restriction fragment length polymorphism (PCR–RFLP) is a technique used to distinguish homologous DNA sequences that can be detected by fragment length difference after DNA samples have been digested with specific enzymes. This method can be used to detect mutations in the *IDH1* gene in a precise, rapid, and inexpensive way (Bujko et al., 2010). 

The rapidity and accuracy of the method in detecting gene mutations in *IDH1* can affect the prognosis, intervention, and survival rate in glioma patients. In Indonesia, tests for IDH mutations are not routinely conducted. Two reasons for this are the limited availability of antibodies suitable for immunohistochemical examinations and, more importantly, the lack of facilities for DNA sequencing. Previous studies have suggested that combining the PCR–RFLP method with DNA sequencing in heterogeneous glioma samples can avoid false negative results and improve cost-effectiveness (Goh et al., 2019). This study aimed to compare IHC, DNA sequencing, and PCR–RFLP in detecting *IDH1* mutation in gliomas. 

## Materials and Methods

*Patients and samples*

This study was approved by the Institutional Review Board (IRB) Universitas Gadjah Mada, Indonesia, with approval number KE/FK/0115/EC/2020. The patients were recruited from Dr. Sardjito Hospital, a tertiary hospital in Yogyakarta Province, Indonesia, and several satellite hospitals in the region. The inclusion criteria were all glioma patients who would undergo tumor removal surgery and agreed to participate in the study. Informed consent was obtained from the patients or from a family member. Tumor tissue samples were obtained in the operating room and stored in the Biobank Facility of Faculty of Medicine, Public Health, and Nursing, Universitas Gadjah Mada, for further processing. All pathological specimens were reviewed and classified by expert pathologists according to The 2016 WHO Classification of Tumors of the Central Nervous System. Demographic and clinical data were collected from medical records.

*DNA extraction*

Genomic DNA was extracted from fresh tumor tissue samples or from formalin-fixed paraffin-embedded (FFPE) tumor tissues from recruited patients. DNA from fresh glioma tissue was extracted using the Quick DNA FFPE MiniPrep Kit (Zymo Research, USA). DNA from FFPE tissue specimens was isolated using the QIAamp DNA FFPE Tissue Kit (QIAGEN, Cat. #56404, Hilden, Germany) according to the manufacturer’s instructions. 

*PCR–RFLP*

Polymerase chain reaction (PCR) was performed using the previously reported mismatched primers to create suitable restriction sites for wild-type sequences (Meyer et al., 2010). Ampliﬁcation with p*IDH1*f-R132 forward (5’- TGGGTAAAACCTATCGAT-3’) and p*IDH1*r-132 reverse (5’-TGTGTTGAGATGGACGCCTA -3’) primers yielded a fragment with a PvuI digestion site in the wild-type sequence (Meyer et al., 2010). 

PCRs were performed in a volume of 20 µL containing 4 µL of genomic DNA, 10 µL of 2xGo Taq green Master Mix, 0.8 µL of each primer, and 4.4 µL of nuclease-free water. The PCR cycling conditions were as follows: initial denaturation at 95^o^C for 2 minutes followed by 30 cycles of denaturation at 95^o^C for 30 seconds, annealing at 55^o^C for 30 seconds, and extension at 72^o^C for 1 minutes. PCR products were digested with the PvuI enzyme and incubated for 1 hour in a 37°C water bath. Then, the electrophoresis was done in 4% agarose gel to see the separation of the bands to identify the *IDH1* gene mutation. 

*DNA sequencing*

PCR to amplify codon R132 of the *IDH1* gene was performed using forward (5’-ACC AAA TGG CAC CAT ACG A-3’) and reverse (5’-GCA AAA TCA CAT TAT TGC CAA C-3’) primers, as reported before (Arita et al., 2014). PCRs were performed in a volume of 25 µL containing 1 µL of each primer, 2 µL of genomic DNA, 3.45 µL of Taq DNA Polymerase (Invitrogen, Thermo Fisher Scientific, Cat. #10342020, Waltham, MA, USA), and 0.2 µL of dNTP (Thermo Scientific, Thermo Fisher Scientific, Cat. #R0191, Waltham, MA, USA). Conditions for PCR cycling were as follows: initial denaturation at 95^o^C for 2 minutes followed by 40 cycles of denaturation at 95^o^C for 30 seconds, annealing at 53^o^C for 30 seconds, and extension at 72^o^C for 2.5 minutes. The PCR products were sequenced using the BigDye Terminator v3.1 Cycle Sequencing Kit (Applied Biosystem, Thermo Fisher Scientific, Cat. #4337455, Waltham, MA, USA). 

*Immunohistochemistry staining*

FFPE samples were cut into 3µm thick slides for immunostaining examination. Next, FFPE sections were incubated, deparaffinized, and rehydrated. Antigen retrieval was done using a decloaking chamber (BioCare Medical, USA). Mouse monoclonal antibody *IDH1* R132H (clone H09) (Dianova GmbH, Germany) was diluted to 1:50 in phosphate buffer saline. Diamino-benzidine for visualization of positive cells was applied, continued with hematoxylin staining as counterstain. Glioma with *IDH1* mutant was used for positive control. 

*IDH1* positive cells were evaluated by 2 experienced pathologists under a light microscope in a high-power field (HPF). The semiquantitative interpretation system by Takano et al. was used (Takano et al., 2011). A tumor cell was considered immunopositive if both the nucleus and the cytoplasm were stained brown. Cases with ≥10% overall positive tumor cells were rated as positive for *IDH1* R132H mutation, while cases with less than 10% overall positive tumor cells were rated as negative for such mutation (Agarwal et al., 2013; Takano et al., 2011)

## Results

*Clinical data*

In total, 61 patients were included in this study, consisting of 34 males and 27 females. The mean age was 44.13-17.13 years old. The majority of patients had WHO grade IV glioma (33 patients, 54.1%), followed by grade III (14 patients, 23%), grade II (13 patients, 21.3%), and grade I (1 patient, 1.6%) (Table 1).

*DNA sequencing*

**Figure 1:**
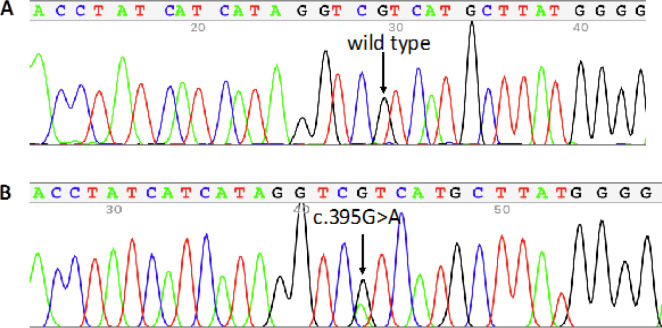
Nucleotide Sequencing Results Showing Wild-Type (A) and Mutant (B) IDH1 Genes

Among 61 patients, 13 (21.3%) of them carried the *IDH1* mutation, as shown by Sanger sequencing (Figure 1). Among this group, 12 were positive for R132H mutation, while 1 patient was found to harbor an R132G mutation. 

*PCR–RFLP*

**Figure 2:**
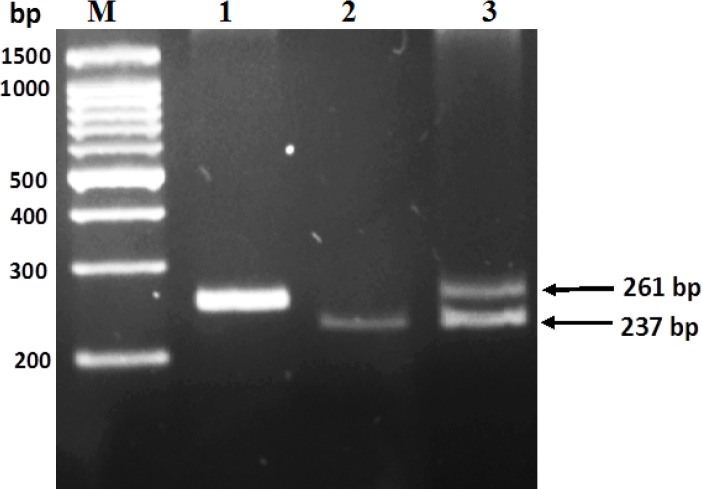
Gel Electrophoresis of PCR–RFLP Product. Fragment of IDH1 gene digested with Pvu1 enzyme. Lane M, 100bp DNA ladder; Lane 1, undigested product; Lane 2, showing one band at 237-bp, indicates a wild type; lane 3, showing double bands at 261-bp and 237-bp, indicates a mutation in codon 132 of the IDH1 gene

PCR amplification of *IDH1* using sequence-specific mismatched primers successfully amplified fragments of the expected size (Figure 2, lane 1). After digestion and separation on agarose gel, the expected RFLP patterns were successfully obtained. Amplification of one band at 237-bp indicates a wild type, while two bands at 261-bp and 237-bp indicate a mutation in codon 132 of *IDH1* gene (Figure 1). 

*Immunohistochemistry detection of IDH1 R132H protein*

**Figure 3:**
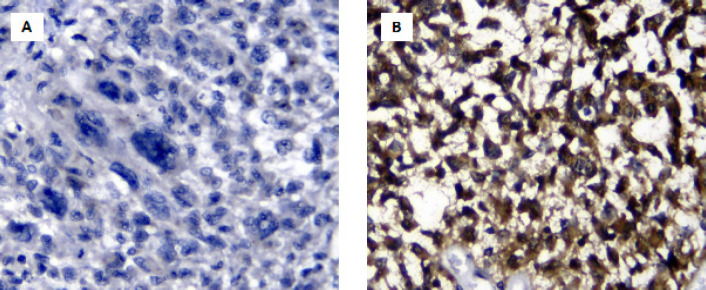
Immunohistochemistry Result of IDH1 R132H (400x). Negative expression is determined as absolutely no reactivity detected (A), or only positively stained in either cytoplasm or nuclei (picture not shown) or in <10% of overall tumor cells. Positive expression is determined as both, nuclei and cytoplasm, are stained brown in >10% of overall tumor cells (B)

*IDH1* R132H immunostaining was found in 13 patients by using H09 Ab speciﬁc for R132H (Figure 3). As expected, one sample with the R132G mutation showed a negative result in IHC using this antibody. Immunostaining was found in the 12 samples with R132H mutation. One of the samples with wild-type *IDH1* gene was also stained.

*Determination of sensitivity, specificity and accuracy*

Concordance of findings between RFLP and DNA sequencing was noted in 100% (61/61) of cases (Tables 1 and 2). Meanwhile, concordance of findings between IHC and DNA sequencing was noted in 96.7% (59/61) cases. One case with discrepancy showed positive *IDH1* R132H staining; however, DNA sequencing and PCR–RFLP showed no such mutation. The other case with discrepancy was shown to have R132G mutation in DNA sequencing. As expected, H09 Ab speciﬁc for R132H was unable to detect this mutation. 

As shown in Table 3, the sensitivity, specificity, positive predictive values (PPV), negative predictive values (NPV), and accuracy for PCR–RFLP were all 100%. Meanwhile, sensitivity and specificity of IHC were 92.3% and 97.9%, respectively, with an accuracy of 96.7%.

**Table 1:** Summary of PCR–RFLP, Gene Sequencing and Immunohistochemical Analyses for Each Tumor Type

			IDH1 mutation					
			PCR–RFLP		IHC		Sequencing	
WHO grade	Pathology	Patients (n)	+	-	+	-	+	-
Grade IV	GBM	33	6	27	7*	26	6	27
Grade III	AA	5	1	4	1	4	1	4
	AO	5	1	4	1	4	1	4
	AOA	1	0	1	0	1	0	1
	AE	3	0	3	0	3	0	3
Grade II	DA	7	2	5	2	5	2	5
	O	1	1	0	1	0	1	0
	OA	2	2	0	1	1*	2	0
	E	3	0	3	0	3	0	3
Grade I	PA	1	0	1	0	1	0	1
Total		61	13	48	13	48	13	48

The results of gene sequencing and RFLP were 100 % congruent. The results of gene sequencing and immunohistochemical analyses were congruent in all but 2 cases (indicated by asterisks). IDH1, Isocitrate Dehydrogenase 1; PCR–RFLP, Polymerase Chain Reaction-Restriction Fragment Length Polymorphism; IHC, Immunohistochemistry; GBM, Glioblastoma; AA, Anaplastic Astrocytoma; AO, Anaplastic Oligodendroglioma; AOA, Anaplastic Oligoastrocytoma; AE, Anaplastic Ependymo Mma; DA, Diffuse Astrocytoma; O, Oligodendroglioma; OA, Oligoastrocytoma; E, Ependymoma; PA, Pilocytic Astrocytoma.

**Table 2:** Results of IDH1 Mutation by IHC Analysis, Direct DNA Sequencing, and PCR–RFLP

			DNA sequencing (n=61)		Total
			IDH1 mutation		
			+	-	
RFLP	IDH1 mutation	+	13	0	13
		-	0	48	48
	Total		13	48	
IHC	IDH1 mutation	+	12	1	13
		-	1	47	48
	Total		13	48	

DNA, Deoxyribonucleic Acid; IDH1, Isocitrate Dehydrogenase 1; IHC, Immunohistochemistry; PCR–RFLP, Polymerase Chain Reaction-Restriction Fragment Length Polymorphism

**Table 3:** Sensitivity, Specificity, and Accuracy of PCR–RFLP and IHC in Detecting IDH1 Mutation Compared to DNA Sequencing as Gold Standard

	Sensitivity (%)	Specificity (%)	PPV (%)	NPV (%)	Accuracy (%)
PCR–RFLP	100	100	100	100	100
IHC	92.3	97.9	92.3	97.9	96.7

DNA, Deoxyribonucleic Acid; IDH1, Isocitrate Dehydrogenase 1; IHC, Immunohistochemistry; NPV, Negative Predictive Value; PCR–RFLP, Polymerase Chain Reaction-Restriction Fragment Length Polymorphism

## Discussion

In our study, we found that 21.3% of the glioma patients carried the *IDH1* mutation. This is consistent with previous studies showing that the frequency of *IDH1* mutations in Asian populations ranges from 7.8% in Japan to 74% in China (Mohamed Yusoff et al., 2016). 

In this study, we showed that PCR–RFLP and DNA sequencing have a concordance value of 100%. Meanwhile, the concordance value between *IDH1* R132H IHC and DNA sequencing was 96.7%. This shows that both PCR–RFLP and *IDH1* R132H IHC have excellent sensitivity and specificity for the detection of *IDH1* mutations in glioma patients.

This finding is consistent with previous studies showing that IHC using specific antibodies for the R132H mutation is a reliable method with high sensitivity and specificity. Several studies have shown a concordance rate between IHC and sequencing for the detection of IDH mutations ranging from 88% to 100% (Zou et al., 2015). In fact, a meta-analysis comparing IHC with DNA sequencing in 1360 cases of glioblastoma showed that the pooled sensitivity and speciﬁcity for *IDH1* IHC were 1.00 (95% CI 0.82–1.00) and 0.99 (95% CI 0.96–1.00), respectively (Pyo et al., 2016). Several studies have even shown that IHC is more sensitive in detecting *IDH1* mutations than is DNA sequencing (Agarwal et al., 2013; Capper et al., 2010; Lee et al., 2013; Preusser et al., 2011; Takano et al., 2011). This is explained by the fact that IHC can detect mutations even when there are only a small number of tumor cells in the sample. However, several other studies have shown the opposite-namely, that sequencing is more sensitive (Catteau et al., 2014; Loussouarn et al., 2012; Mellai et al., 2011). In these studies, the reason stated is that the antibodies used can detect only R132H mutations and not other mutations, such as R132C, R132L, R132S, and R132G (Zou et al., 2015). We confirmed this result in our study. Antibodies for proteins with the R132H mutation showed negative results in one sample with the R132G mutation. 

Besides having high accuracy, immunohistochemical examination also has other advantages. IHC is relatively easy to perform in most pathology labs, is time- and space-efficient, is relatively inexpensive, and can evaluate the morphological expression patterns of mutated proteins. However, the weakness of this test is that it cannot detect mutations other than the R132H mutation (Catteau et al., 2014; Preusser et al., 2011). That is why the existing algorithm recommends that in patients with negative *IDH1* R132H staining in the IHC examination, DNA sequencing should be performed. However, as explained before, DNA sequencing is labor-intensive, requiring sophisticated equipment and trained personnel, and is not readily available in all neuropathological centers (Agarwal et al., 2013; Catteau et al., 2014). Therefore, in this study, we proposed the PCR–RFLP-based approach for *IDH1* mutation detection after negative IHC results, instead of DNA sequencing. 

The *IDH1* gene does not have a suitable location for the action of restriction endonuclease. We performed PCR using sequence-specific mismatched primers, as previously reported (Meyer et al., 2010). This PCR introduced the PvuI digestion enzyme restriction site in the wild-type sequence. As a result, the two alleles in patients with wild-type *IDH1* sequence will be cleaved, while in patients with mutations in codon 132, one of the alleles cannot be cleaved, resulting in two signals in the agarose gels (Goh et al., 2019; Meyer et al., 2010)

Our study showed that this PCR–RFLP had 100% concordance with DNA sequencing. This shows that PCR–RFLP is a reliable method for detecting IDH mutations. This finding is consistent with previous studies (Bujko et al., 2010; Mohamed Yusoff et al., 2016), indicating that PCR–RFLP is a sensitive and specific method in detecting *IDH1* mutation in codon 132.

Compared to IHC, PCR–RFLP is not prone to errors due to subjective microscopic observation. Compared to other molecular methods, such as DNA sequencing and real-time PCR, PCR–RFLP also has several advantages. This method is faster, cheaper, easier to do, and does not require expensive additional equipment such as DNA sequencers or real-time PCR machines (Bujko et al., 2010). These characteristics facilitate the adaptation of PCR–RFLP examinations in countries with limited resources such as Indonesia.

The weakness of the PCR–RFLP examination is generally the same as that of other PCR-based examinations. The sensitivity of this method is greatly influenced by the quality of the samples obtained. At least 50% of the cells in the sample must be tumor cells. This is a problem because gliomas are infiltrative tumors that often mix with normal cell populations. False negative results can be obtained if the amount of tumor DNA obtained is insufficient due to the small biopsy size, extensive necrosis, or mixing with normal tissue (Agarwal et al., 2013; Catteau et al., 2014; Lee et al., 2013). Therefore, we argue that PCR–RFLP should be used in addition to IHC examination, not as a replacement for it. 

The current recommendation for IDH mutation detection is *IDH1* R132H mutation identification using IHC, followed by DNA sequencing if IHC shows negative results. Based on the results in our study, we recommend that, in resource-limited settings, the PCR–RFLP-based approach for *IDH1* mutation detection, rather than DNA sequencing, be used after negative IHC results. The downside of this approach is the inability of the PCR–RFLP method in our study to identify types of missense mutation in codon 132 (R132H mutation or others), or mutations in other locations in the *IDH1* gene. However, because the vast majority of mutations in the *IDH1* gene occur at this location, and most are mutations of the R132H type, we do not consider this a significant problem in clinical practice.

In conclusion, we showed that both PCR–RFLP and IHC have high accuracy in detecting *IDH1* mutation. We recommend the combination of *IDH1* R132H IHC and PCR–RFLP for *IDH1* mutation detection in resource-limited settings.
